# Persistent viremia by a novel parvovirus in a slow loris (*Nycticebus coucang*) with diffuse histiocytic sarcoma

**DOI:** 10.3389/fmicb.2014.00655

**Published:** 2014-12-01

**Authors:** Marta Canuti, Cathy V. Williams, Sashi R. Gadi, Maarten F. Jebbink, Bas B. Oude Munnink, Seyed Mohammad Jazaeri Farsani, John M. Cullen, Lia van der Hoek

**Affiliations:** ^1^Laboratory of Experimental Virology, Department of Medical Microbiology, Center for Infection and Immunity Amsterdam (CINIMA), Academic Medical Center of the University of AmsterdamAmsterdam, Netherlands; ^2^Duke Lemur Center, Duke UniversityDurham, NC, USA; ^3^Department of Population Health and Pathobiology, College of Veterinary Medicine, North Carolina State UniversityRaleigh, NC, USA; ^4^Department of Virology, Tehran University of Medical SciencesTehran, Iran

**Keywords:** parvovirus, virus discovery, slow loris, *Nycticebus coucang*, histiocytic sarcoma, VIDISCA, hematopoietic tumor, oncovirus

## Abstract

Cancer is one of the leading health concerns for human and animal health. Since the tumorigenesis process is not completely understood and it is known that some viruses can induce carcinogenesis, it is highly important to identify novel oncoviruses and extensively study underlying oncogenic mechanisms. Here, we investigated a case of diffuse histiocytic sarcoma in a 22 year old slow loris (*Nycticebus coucang*), using a broad spectrum virus discovery technique. A novel parvovirus was discovered and the phylogenetic analysis performed on its fully sequenced genome demonstrated that it represents the first member of a novel genus. The possible causative correlation between this virus and the malignancy was further investigated and 20 serum and 61 organ samples from 25 animals (*N. coucang* and *N. pygmaeus*) were screened for the novel virus but only samples collected from the originally infected animal were positive. The virus was present in all tested organs (intestine, liver, spleen, kidneys, and lungs) and in all banked serum samples collected up to 8 years before death. All attempts to identify a latent viral form (integrated or episomal) were unsuccessful and the increase of variation in the viral sequences during the years was consistent with absence of latency. Since it is well known that parvoviruses are dependent on cell division to successfully replicate, we hypothesized that the virus could have benefitted from the constantly dividing cancer cells and may not have been the cause of the histiocytic sarcoma. It is also possible to conjecture that the virus had a role in delaying the tumor progression and this report might bring new exciting opportunities in recognizing viruses to be used in cancer virotherapy.

## Introduction

The Sunda slow loris (*Nycticebus coucang*), a Strepsirrhine primate which belongs to the family Lorisidae, is a nocturnal, arboreal prosimian species native to Indonesia (Sumatra), western Malaysia (Peninsular Malaysia), Singapore and southern Thailand (Nekaris and Streicher, 2008). Slow loris, listed as a vulnerable species by the International Union for Conservation and Nature (IUCN) (IUCN Red List of Threatened Species, available at: www.iucnredlist.org), are protected by law in Malaysia, Thailand, and Indonesia since their conservation status is a matter of concern. The biggest threat endangering those animals is the pet trade—they are the most commonly protected primates species sold as exotic pets in southeast Asia (Nekaris and Nijman, 2007), but habitat loss and the fact they are killed as crop pests also jeopardize their survival (Nekaris and Streicher, 2008). Because of these reasons a better understanding of the causes and the dynamics of diseases in *N. coucang* is advantageous for conservation attempts and captive management of this species (Remick et al., 2009). In addition, valuable information can be gathered by the study of diseases in non-human primates, which in turn might be beneficial for human health.

We investigated a case of histiocytic sarcoma (HS) in a *N. coucang* identified at the Duke Lemur Center (DLC) in Durham, North Carolina. HS is a highly aggressive hematopoietic tumor defined as a malignant proliferation of cells showing morphological and immunophenotypic features of mature tissue histiocytes, cells of the innate immune system derived from bone marrow monocytes which differentiate into dendritic cells and tissue localized macrophages (Fulmer and Mauldin, 2007; Takahashi and Nakamura, 2013). Tumors can be localized or disseminated with lymph nodes being the most common site of proliferation, followed by organs of the gastrointestinal tract, spleen, soft tissues, and skin (Takahashi and Nakamura, 2013). It is a rare type of cancer in humans and cases have been reported in other species of animals, including chickens, dogs, cats, camels, macaques, and lemurs (Fulmer and Mauldin, 2007; Friedrichs and Young, 2008; Soshin et al., 2008; Molenaar et al., 2009; Remick et al., 2009; Takahashi and Nakamura, 2013). Although the cause of HS is largely unknown, a viral etiology can be postulated as the existence of viruses involved in the development of hematopoietic cancers have been recognized, as in the case of Epstein Barr and human T-lymphotropic virus-induced human lymphomas and similar viruses in non-human primates (Miller et al., 1972; Donahue et al., 1992; Feichtinger et al., 1992; Vereide and Sugden, 2009; Qayyum and Choi, 2014). Additionally it has been proven that the subgroup J avian leukosis virus is associated with the development of histiocytic sarcomatosis in chickens (Pandiri et al., 2009) and an association between persistent Epstein Barr virus infection and human HS has been reported (Kramer et al., 1985). In this study we applied a sequence independent virus discovery technique combined with high throughput sequencing, VIDISCA-454 (De Vries et al., 2011), to investigate the possible involvement of a previously unknown virus in the development of HS in a *N. coucang*. Several sequences with homology to parvoviral genes were identified indicating the presence of a novel parvovirus, whose genome was subsequently fully sequenced.

Parvoviruses (viral family *Parvoviridae*) are small non-enveloped single stranded DNA viruses which are able to infect a wide range of species of vertebrates (subfamily *Parvovirinae*) and arthropods (subfamily *Densovirinae*). According to the latest ICTV classification (2013) 8 genera are recognized within the subfamily *Parvovirinae* and 5 of them include viruses infecting primates (Cotmore et al., 2013; Cotmore and Tattersall, 2014). Some parvoviruses classified within the genus *Dependoparvovirus* need the presence of a helper virus for a productive infection while viruses within other genera—the so called autonomous parvoviruses—are S phase–dependent: cells must undergo the S phase of growth for viral replication to occur (Berns, 1990).

The spectrum of parvovirus induced diseases, which mainly involve young individuals, is very wide and varies from more severe forms [like severe enteritis with high mortality in young dogs (Goddard and Leisewitz, 2010), erythema infectiosum or hydrops fetalis in children both caused by parvovirus B19 (Heegaard and Brown, 2002, p. 19), or the Aleutian disease in minks (Best and Bloom, 2005)] to milder forms [like common respiratory and gastrointestinal diseases in humans (Jartti et al., 2012)]. Finally, parvovirus infections can also occur in asymptomatic individuals (Heegaard and Brown, 2002, p. 19; Lau et al., 2008; Clegg et al., 2012). Although there is no formal proof of the existence of oncoviruses within this family, parvoviruses have been reported in literature to be associated with both solid and hematopoietic cancers (Fisgin et al., 2002; Li et al., 2012; Schildgen et al., 2013; Ibrahem et al., 2014). However, since they rely on actively replicating cells, the increased presence of these viruses in individuals with cancer might also derive from the permissive nature of the tumor cells and some of these viruses have even proven to possess oncosuppressive effects on transformed cells (Berns, 1990; Nüesch et al., 2012).

Besides reporting the discovery and the molecular characterization of a novel parvovirus, the scope of this study was to determine whether a possible correlation existed between this virus and the presence of the histiocytic sarcoma in a *N. coucang*.

## Materials and methods

### Clinical case

The virus was identified in a 22 year old, male *Nycticebus coucang* (slow loris) named Buddha, held in captivity for 22 years at the Duke University Primate Center and which had no prior significant health issues. The individual was diagnosed with neoplasia and euthanized due to poor condition, although a routine physical inspection 8 months before death revealed an enlarged spleen. A complete postmortem examination was performed.

### Clinical samples

Representative tissues of Buddha were collected and fixed in 10% neutral formalin. Fixed tissue was processed routinely into paraffin blocks and sections were stained with hematoxylin and eosin and reviewed by a board of certified veterinary pathologist.

Twenty serum samples (belonging to 16 individuals: 11 *N. coucang* and 5 *Nycticebus pygmaeus*) and 61 organs collected at necropsy (belonging to 17 individuals: 11 *N. coucang*—including Buddha—and 6 *N. pygmaeus*) were screened for the presence of the virus. Organ samples included 17 livers, 6 spleens, 15 kidneys, 10 lungs, 6 hearts, 7 intestines (5 small intestines and 2 large intestines). Altogether these samples belonged to 25 individuals (18 *N. coucang* and 7 *N. pygmaeus*) with various types of disease. Liver, spleen, kidney, lung, and intestine samples from Buddha were available as well as serum or whole blood collected on 4 different time points: year 2000 (serum), year 2005 (serum), year 2007 (whole blood), and year 2008 (serum).

Serum samples and organs were stored at −80°C until virological examinations were performed.

Housing management and sample collection from the animals in this report were approved by the appropriate federal and institutional regulatory authorities.

### Virus discovery

Sequence independent virus discovery was performed on a serum sample collected from Buddha at necropsy (2008) with the previously described VIDISCA-454 procedure (De Vries et al., 2011) with minor modifications. After a centrifugation and DNase treatment to remove both intact cells and host DNA derived from broken cells, viral nucleic acids were isolated from 100 μl of serum with the QIAmp DNA mini kit (Qiagen). Subsequent to the ligation of adaptors containing specific Roche-454 primer binding sequences, an amplicon size-selection was performed to prevent the amplification of DNA fragments smaller than 200 bp using Agencourt AMPure XP beads (Beckman Coulter), followed by 30 cycles of PCR amplification. The amplified library was subjected to 2 consecutive purification rounds with Agencourt AMPure XP beads to completely remove excess primers and short fragments and DNA concentration was measured on a Qubit Fluorometer (Quant-it ds DNA HS kit, Invitrogen). The library was pooled with other samples, the average size of the whole library was estimated with Agilent 2100Bioanalyzer (high sensitivity DNA kit, Agilent Technologies) and the final concentration (copies/μl) was calculated using the KAPA Library Quantification kit (KAPA Biosystems). Samples were then diluted to a final concentration of 1 million copies/μl and used as input for the emulsion PCR (LIB-A emPCR kit, Roche) and 454 pyrosequencing (Roche).

After sequencing, primer sequences were trimmed from every read and sequences were assembled with CodonCode Aligner software, version 3.5.6. The contigs (consensus sequences derived from reads found multiple times) and unassembled sequences were compared to known nucleotide and protein sequences in the GenBank database using different standalone BLAST tools (blastn and tblastx) (Altschul et al., 1990). Blast results were visualized using the MEGAN software version 4.70.4 (Huson et al., 2011).

### Full genome sequencing

Specific primers were designed on the viral sequences identified with VIDISCA-454 and PCRs using DreamTaq DNA polymerase (Thermo Scientific) were performed to connect fragments. Sequencing reactions were carried out with nested primers directly on the amplified products using the Big Dye terminator chemistry (BigDye^®^ Terminator v1.1 Cycle Sequencing Kit, Applied Biosystems).

The ends of the genome were determined using genomic fragments obtained by specific digestion with 2 different restriction enzymes (MseI and CviAII from New England Biolabs) to obtain overlapping fragments, to which VIDISCA adaptors were subsequently ligated; semi specific PCRs were then performed with a combination of one primer annealing to the known viral sequence and one to the adaptor. After sequencing the obtained amplicons, the novel sequence was used as a template for new primer design and the whole procedure was repeated until reaching the end of the genome. Specific PCRs were used as confirmation. All primers used for PCR and sequencing reactions are available upon request.

### Viral screening and DNA quantification

DNA isolations were performed from 100 μl of serum with the QIAmp DNA mini kit (Qiagen) and from 40 μl of blood or about 20 mg of tissue (about 5 mg for spleen) with the DNeasy Blood and Tissue kit (Qiagen). Absolute quantification of viral DNA (copies/ml of serum or blood and copies/g of tissue) was achieved using plasmid-based standards: a portion of the NS1 ORF (position in the complete genome: nt 1129–1527) was amplified and cloned into TOPO^®^ cloning vector according to the instructions of the manufacturer (TOPO^®^ TA cloning, Invitrogen), followed by plasmid purification (Plasmid DNA purification Nucleobond Xtra Midi, Macherey-Nagel) and quantification (NanoDrop 2000c, Thermo Scientific). Quantitative Real Time PCR assays were performed with IQ Supermix (BioRad) and using 4 μl of DNA as input, with the following primers and probe: Buddha_RT_F, GCTAATCTGGTGGGAAGAAGG; Buddha_RT_R, CCTTTGCGATCTACCCTGAC; Buddha_RT_P, 5′FAM—CCGCCAAGGAGAGCCTTAGCAC—TAMRA 3′. Real-time PCR reactions were performed with the Light Cycler 480 system (Roche).

To detect possible differences between sequences obtained from various organs or different years, all positive samples were subjected to a specific amplification of a 1231 nt long portion of the VP1 gene using DreamTaq DNA polymerase (Thermo Scientific) with primers Buddha_VP_5F—ATGTCTCCACTCATTCTGGTG—and Buddha_14_R—ACGATCTGGGTAGATGACTTC. Amplicons were diluted 1:10 and directly sequenced employing the Big Dye terminator chemistry (BigDye^®^ Terminator v1.1 Cycle Sequencing Kit, Applied Biosystems).

### Identification of integrated or episomal viral DNA

**Figure 1:**
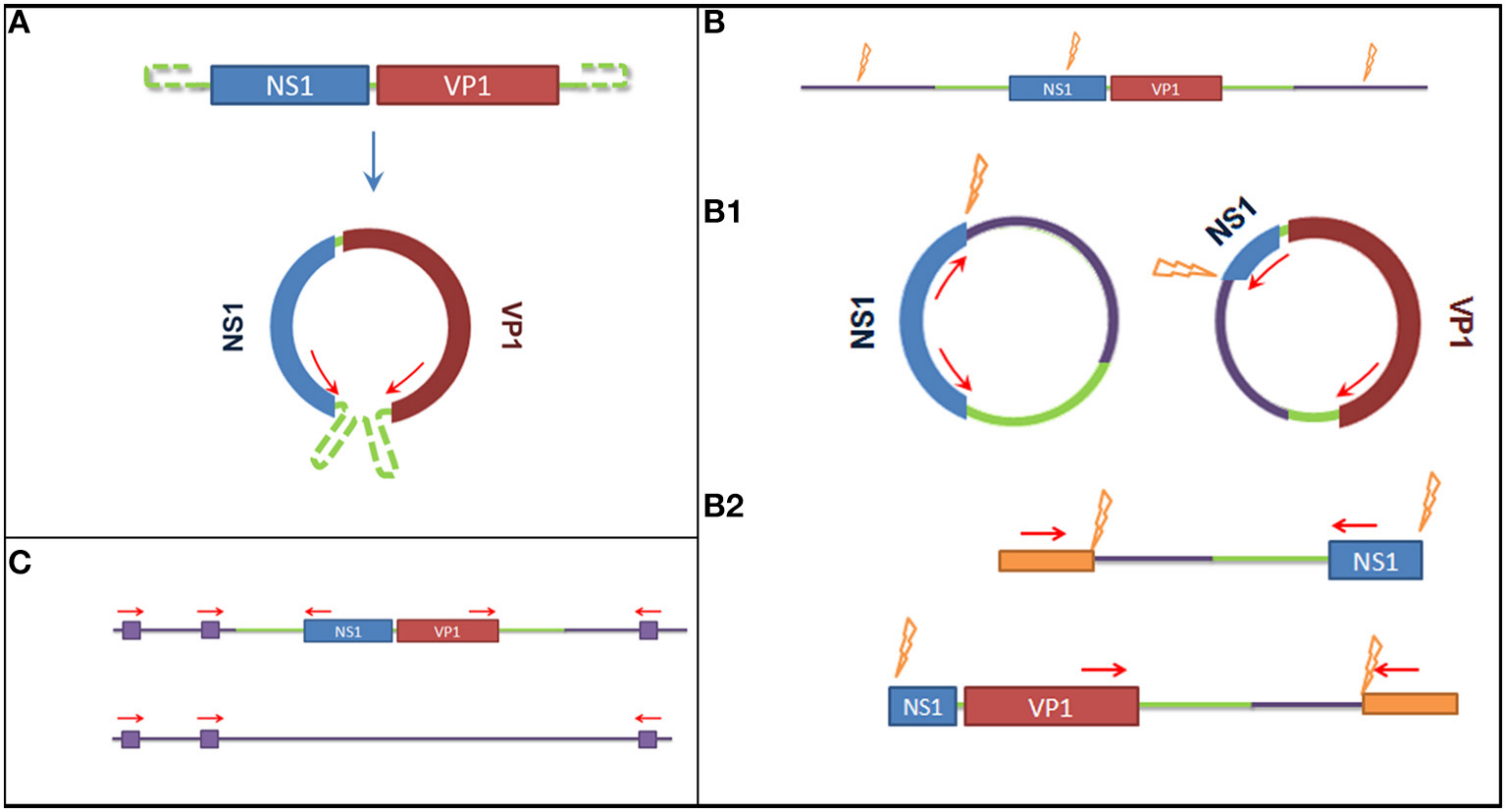
**Graphical overview of the methods used to detect latent viral forms. (A)** Method used to detect episomal genomic DNA. On the top the genome organization of the Sl.L-PV-1, on the bottom a representation of the predicted circular form. Red arrows represent primer binding sites. The use of reverse primer annealing at the beginning of NS1 and forward primers annealing at the end of VP1 allows the amplification the connecting genomic sequence. **(B)** Restriction enzyme based methods to detect integrated virus. The purple lines represent the host genomic sequence, the orange flashes indicate restriction enzyme sites and the orange boxed indicate artificially ligated adaptors. **(B1)** It is shown how, after overnight ligation of the digested DNA, the obtained fragment would self-ligate in a circular form and how a specific amplification would allow to amplify the integration site. **(B2)** It is shown how, after the ligation of specific adaptors to the obtained digested fragments, a semi specific PCR would allow the amplification of the integration site. **(C)** Method to detect the integrated virus based on repeated Alu sequences in the host genome. Purple boxes represent Alu sequences and red arrows represent primer binding sites. A comparison of the PCR results obtained amplifying the template with either a mixture of Alu binding primers and specific primers or Alu binding primers alone (negative control) would allow to amplify the integration site and determine which amplified fragment on gel corresponds to it.

To detect the presence of latent viral forms (extra-chromosomal circular episomal DNA or integrated DNA) in the different tissues, specific PCR-based assays were designed. A graphical overview of the methods used to detect latency is available in Figure 1. For this purpose the DNA isolated from all organs collected during the necropsy of Buddha (lung, liver, spleen, kidney, small, and large intestine) and from blood (collected one year before the animal died) were used as input.

To detect the presence of circularized viral genome an inverted nested PCR was performed using primers annealing at the end of the two open reading frames (ORFs) (Figure 1A): I_Buddha_R1—CCTTGTTCGATCTGTCCAAAATAATTGC—and I_Buddha_F1—CCAGTAGTGGAGAAGTCATCTACCC—for the first step of amplification; I_Buddha_R2—GTAATTGCTTTAATGGCTTTGATTCCCAG—and I-Buddha_F2—TGGAAGTCGAGTAAAATACCGACACATG—for the nested amplification. As a control DNA isolated from serum samples (collected in 2008 both with and without VIDISCA pre-treatment) was also included.

To detect chromosome-integrated viral forms 3 different approaches have been employed, of which 2 involved a specific enzymatic digestion of the isolated DNA (Figure 1B): 7.5 μl DNA eluate was cut (restriction site at nucleotides 868–873) in a 20 μl reaction mix containing 5 U of NdeI restriction enzyme (New England Biolabs) during an incubation of 2 h at 37°C. Prior to inactivation, 5 μl of cut DNA were collected and used to perform a specific ligation of VIDISCA adaptors (De Vries et al., 2011) to the digested fragments using 4 U of T4 DNA ligase (life technologies). Afterwards 2 semi virus-specific nested amplifications were performed using one primer complementary to the virus (I_Buddha_R1 and I_Buddha_R2 for the 5′ side; I_Buddha_F1 and I_Buddha_F2 for the 3′ side) and one adaptor specific primer. In parallel, 5 μl of the cut DNA was subjected to an inactivation step for 20 min at 65°C, followed by an overnight incubation at room temperature with 4 U of T4 DNA ligase to allow self-ligation and consequent circularization of the digested fragments (containing host sequences flanking the viral integrated DNA). Two specific inverted nested PCRs were used afterwards with primer pairs I_Buddha_F1/I_Buddha_R3 (TCCCGACACAAATAATCGGACAACC) and I_Buddha_R1/I_Buddha_F3 (GACGTGCTTTGTTAGAATCTGTTCCTG) during the first step and I_Buddha_F2/I_Buddha_R4 (GAAATTTGAATTTCCATCTTAGCTTGAGTC) and I_Buddha_R2/I_Buddha_F4 (CAAACCCTGCAATGGTGTGTAGATG) during the second amplification step.

The third approach for integrated DNA detection made use of the Alu elements, DNA sequences which are highly repeated in primate genomes (Häsler and Strub, 2006). A primer (Ny_Alu: CCTCCCAGAGTGCTAGGATTGCAC) binding to one of these region was designed (according to GenBank sequence with accession number DQ822059) and used alone (as a negative control) or in combination with viral specific primers (I_Buddha_R1 or I_Buddha_F1 for the first amplification step and I_Buddha_R2 or I_Buddha_F2 for the nested amplification) to amplify the putative integration region (Figure 1C).

All amplifications were performed by using 5 μl of DNA as input in a 50 μl PCR mix containing DreamTaq DNA polymerase (Thermo Scientific) and 0.2 μM primers: PCRs were performed according to the following thermo profile: initial denaturation at 95°C for 5 min, followed by 35 cycles (25 during the nested PCR) at 95°C for 30 s, 60°C for 30 s and 72°C for 1.5 min (for the episomal DNA test) or 3 min (for the integration tests) and a final elongation cycle at 72°C for 7 min. Positive PCR products were cloned into the TOPO^®^ cloning vector (TOPO^®^ TA cloning, Invitrogen) and sequenced employing Big Dye terminator chemistry (BigDye^®^ Terminator v1.1 Cycle Sequencing Kit, Applied Biosystems) using betaine at a final concentration of 1 M to resolve secondary structures in the DNA.

### Sequence and phylogenetic analysis

To characterize the phylogenetic placement of the novel virus, 38 NS1 amino acid sequences of known parvoviruses were downloaded from the GenBank database and aligned together with the one predicted in this study from the obtained nucleotide sequence using the Cobalt Multiple Alignment Tool (http://www.ncbi.nlm.nih.gov/tools/cobalt/). Only complete sequences were included in the analysis. Alignments were manually edited when needed with BioEdit software version 7.0.5.3 (Hall, 1999) and then used for phylogenetic inference. A model selection was performed to identify the best model for distance estimation and phylogenetic trees were constructed with Mega software version 6.06 (Tamura et al., 2013) using the Maximum-likelihood method (Felsenstein, 1981). To test the robustness of the analysis a bootstrap analysis (1000 replicates) (Felsenstein, 1985) was performed and only clusters associated with a value higher than 75% were considered significant.

To identify possible mutation between viral sequences amplified from different tissues or different time points, nucleotide sequences were aligned using ClustalX version 2.1 (Larkin et al., 2007) and identities between sequences were calculated with BioEdit software version 7.0.9.0 (Hall, 1999).

DNA secondary structure predictions were obtained using the mfold web server (http://mfold.rna.albany.edu/?q=mfold/download-mfold).

### Accession numbers

The genomic sequence obtained in this study has been deposited in the GenBank database under the accession number KP120516.

## Results

We examine here the case of Buddha, a male slow loris, who was euthanized at the age of 22 years because of diffuse HS. A serum sample collected at necropsy was used as input for virus discovery in search for a novel virus that could explain the disease.

### Pathological details

During the postmortem examination the principal observations included a significantly enlarged spleen with a homogeneous cut surface and a mottled liver with irregular patches of pallor. Pancreatic and mediastinal lymph nodes were enlarged.

Incidental lesions included a cystic mass in the right mammary gland and several small ulcers in the fundus of the stomach. Histologic examination of the spleen revealed a loss of normal architecture and an intense infiltration of neoplastic round to polygonal cells with moderate variation in cell and nuclear sizes (anisocytosis and anisokaryosis). Mitotic figures were common and multinucleate cells were occasionally seen. In the liver there were similar neoplastic cell aggregates surrounding the portal veins and the central veins, expanding the associated connective tissue. Ingested red blood cells (erythrophagocytosis) by neoplastic cells were also observed. Affected lymph nodes were characterized by a loss of normal architecture which was replaced by sheets of round to polygonal neoplastic cells similar to those found in the liver and spleen. These findings led to a diagnosis of disseminated histiocytic sarcoma.

Additional findings included evidence of an apocrine gland cyst causing the mass in the right mammary gland. In addition there was evidence of inflammation and necrosis in the adrenal glands associated with a protozoal infection, interpreted to be Toxoplasma gondii. In addition, large deeply basophilic intranuclear inclusions were found in a minority of adrenal cortical cells. Renal tubules contained eosinophilic droplets in the epithelial cells, as is often seen in mice with histiocytic sarcoma, although the cause is not known. Chronic interstitial nephritis was also evident.

### Identification of a novel parvovirus and complete genome sequencing

To explore the possibility of viral involvement in the etiopathogenesis of the HS we used VIDISCA-454, a sequence independent virus identification technique which is able to detect virtually any DNA and RNA virus from various clinical samples (van der Hoek et al., 2004; Canuti et al., 2011; Jazaeri Farsani et al., 2013; Tan et al., 2013; Canuti et al., 2014; Oude Munnink et al., 2014; Pariani et al., 2014). A total of 8003 sequence reads were obtained from the serum sample of the slow loris with HS and, among these, 5 sequences were identified with homology to different members of the *Parvovirinae* subfamily: 3 were identified with blastn (nucleotide identity: 62–70%) and 2 were only recognizable when identity search was performed at amino acid level (amino acid identity: 69–82%). Those recognized as viral fragments were used as template for primer design, and a combination of specific PCRs and genome walking techniques allowed retrieval of the almost complete genomic sequence of the virus (only part of the terminal repeats is lacking), which was tentatively named Slow Loris parvovirus 1 (Sl.L-PV-1). The genomic sequence allowed us to identify 3 further viral sequences which were not identifiable via identity search. In total 35 reads (0.44% of the total) belonged to the novel virus.

### Genome organization

The rules set by the parvovirus study group in the latest ICTV report mention that a novel parvovirus, which lacked isolation, can be classified within the *Parvoviridae* family only if the complete coding sequence is provided and the typical parvoviral motif patterns can be recognized (Cotmore et al., 2013).

**Figure 2:**
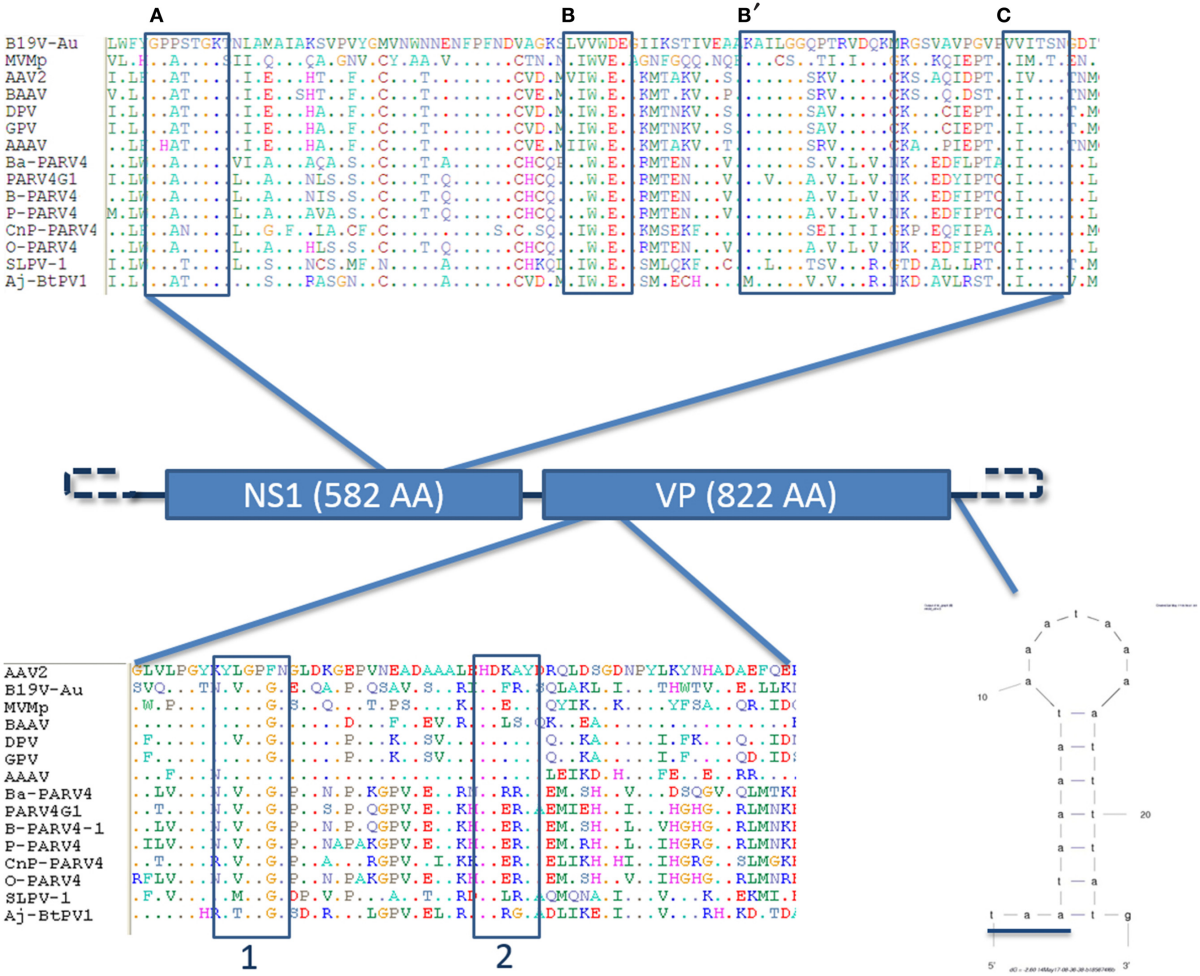
**Genome organization of the slow loris parvovirus 1 (Sl.L-PV-1)**. Schematic representation of the genome organization of Sl.L-PV-1 with the two main ORF (NS1 and VP1) indicated with the respective predicted protein size. On the top the helicase domain of Sl.L-PV-1 (AA 336–423) is compared with the one of other members of the *Parvovirinae* sub-family. The conserved Walker motifs **(A,B,B',C)** are indicated in boxes (Walker et al., 1997; Lou et al., 2012). For sequences accession numbers see Figure 3. The phospholipase A2 domain comparison between Sl.L-PV-1 (AA 109–167) and other members of the *Parvovirinae* sub-family is presented at the bottom. The conserved Calcium binding domain (1) and enzymatic core (2) motifs are indicated by boxes (Zádori et al., 2001). Finally, the hypothetical 5′ and 3′ specific terminal hairpins are represented by dashed lines and on the right a small hairpin identified only on the 3′end of the genome is shown just after the underlined VP1 stop codon. The mfold web server (http://mfold.rna.albany.edu/?q=mfold/download-mfold) was used to predict the secondary structure.

The genome of the novel parvovirus is 4844 nt and the genomic organization reflects the other members of the *Parvovirinae* subfamily with 2 large ORFs and terminal sequences which form end loop structures important for viral replication, although the structure of the loops could not be entirely resolved since only partial sequences could be retrieved (Figure 2) (Berns, 1990). The first ORF—located at the 5′ part of the genome (nt 146–1894)—putatively encodes for the non-structural protein NS1 (582 AA). The second ORF—located at the 3′ part (nt 2187–4655)—encodes for the capsid protein VP1 (822 AA).

Other molecular features typical of parvoviruses were also identified (Figure 2). A phospholipase A_2_ domain of Sl.L-PV-1, that is conserved in the majority of parvoviruses and which is essential for viral genome transfer to the nucleus, is located at the N-terminal part of the VP1 unique region and contains the typical PLA_2_ catalytic domain (HDXXY, AA 140–144) and Ca^++^ binding loop (YXGXG, AA 117–121) (Zádori et al., 2001).

Another molecular marker of parvoviruses is the presence of conserved helicase sequence motifs in the NS1 protein (Walker et al., 1997; Lou et al., 2012). Within the carboxy-terminal half of the NS1 protein of Sl.L-PV-1 (AA 336-423) the typical ATP binding loop or p-loop (Walker box A: GXXXXGK(T/S)) could be identified, immediately followed by the Mg++ binding Walker B (hhhh(D/E)E) and B' motifs. Finally, the C motif (a stretch of hydrophobic residues usually followed by asparagine), common to the helicases belonging to the superfamily type III, was also present (Figure 2).

**Figure 3:**
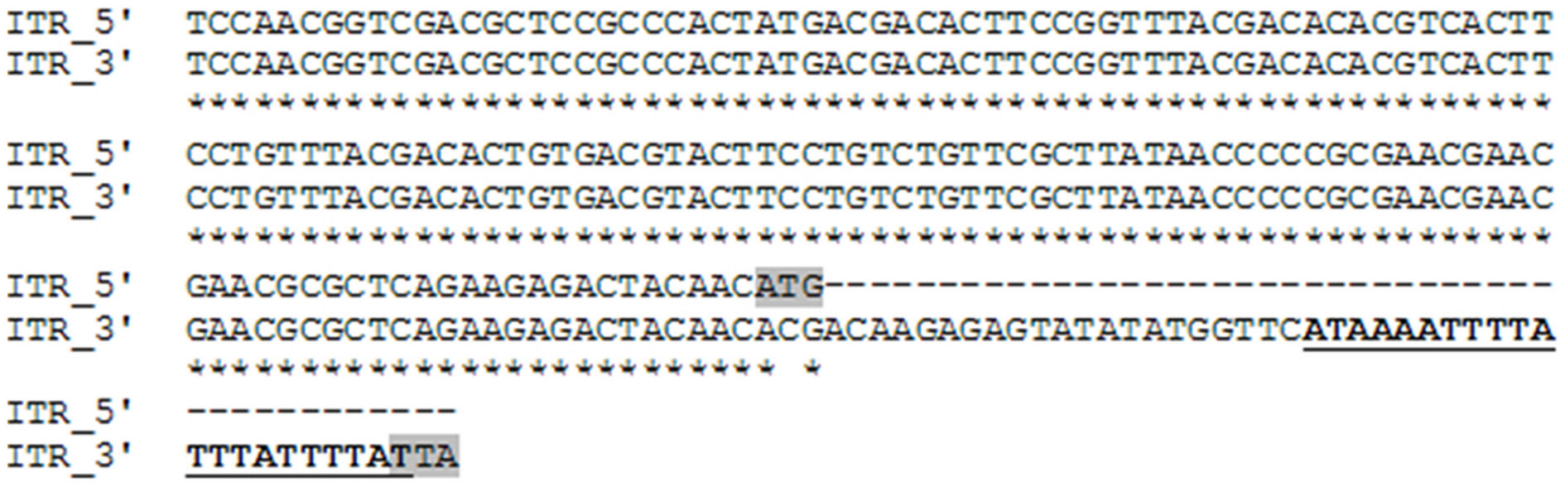
**Alignment of the available inverted terminal repeat sequences of Slow loris parvovirus 1 (Sl.L-PV-1)**. The 5′ side ITR is oriented 5′ to 3′, while the 3′ side ITR is oriented in the opposite direction. The start codon of NS1 as well as the stop codon of VP1 are highlighted in gray. The sequence of the small hairpin unique to the 3′ side non-coding region is underlined and in boldface.

We were able to obtain only part of the sequences from the terminal non coding regions, 148 and 192 nt of the 5′ and 3′ end respectively. An alignment of these 2 sequences shows that they are 100% identical (but oriented in the opposite direction) and start differentiating from the ATG start codon of NS1, which becomes ACG on the 3′ side terminal sequence (Figure 3). These data suggest that Sl.L-PV-1 possesses identical inverted terminal repeats. Besides, a 44 nt sequence was identified which is present only on the 3′ side of the genome, just after the TAA stop codon of VP1/VP2, that contains a 21 nt region that can fold into a small hairpin (Figures 2, 3).

### Phylogenetic analysis and proposed classification

**Figure 4:**
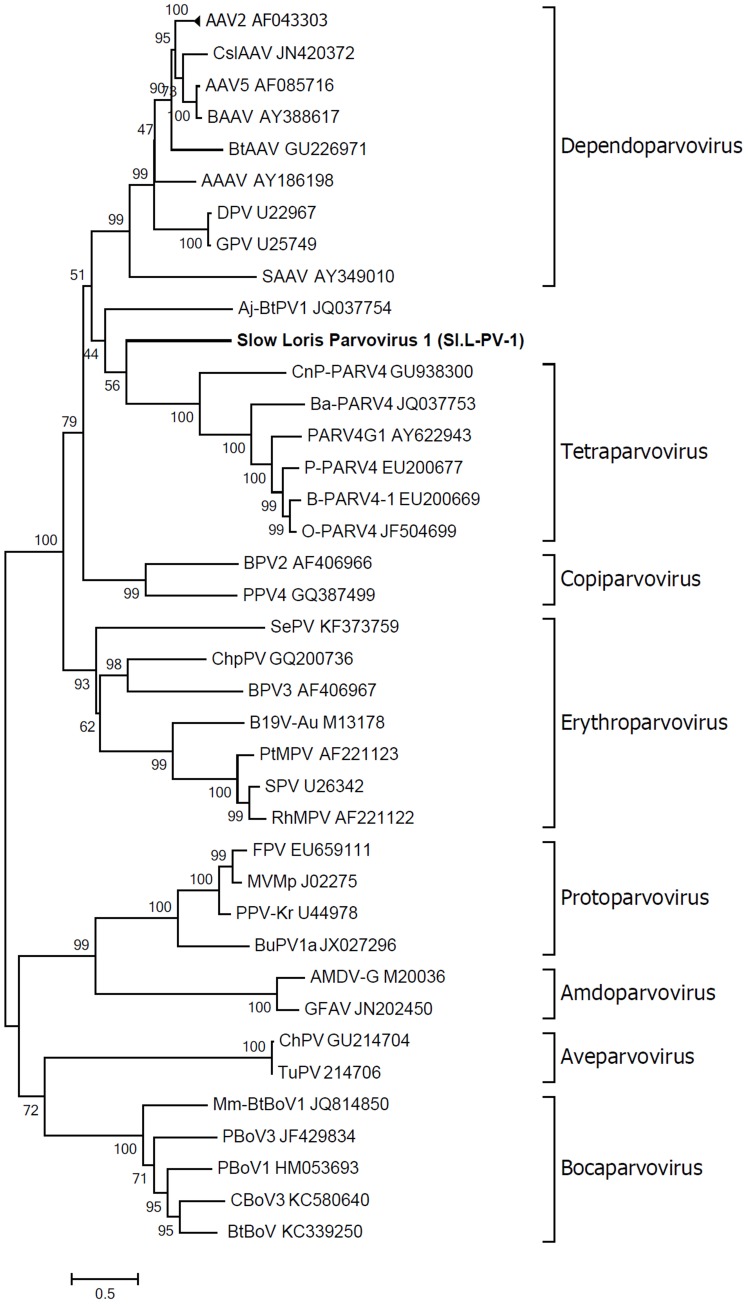
**Phylogenetic analyses of slow loris parvovirus 1 (Sl.L-PV-1)**. The evolutionary history of Sl.L-PV-1 and the other members of the *Parvovirinae* subfamily was inferred using the Maximum Likelihood method (Felsenstein, 1981) based on the rtREV + Freq model (Dimmic et al., 2002), identified as the best fitting model after the model test analysis, using MEGA6 (Tamura et al., 2013). A distance Gamma distribution was used to model evolutionary rate differences among sites (+G, parameter = 2.5202). The rate variation model allowed for some sites to be evolutionarily invariable ([+*I*], 4.0614% sites). All positions with less than 95% site coverage were eliminated. The outcome of the bootstrap analysis (Felsenstein, 1985) is shown next to the branches. The genera are designated by square brackets and the accession numbers of the reference strains are indicated next to the strain name. The virus described in this study is indicated in bold.

Phylogenetic analysis of Sl.L-PV-1 was performed using the predicted amino acid sequences of the NS1 protein belonging to members of the *Parvovirinae* subfamily (Figure 4). In the phylogenetic tree the 8 *Parvovirinae* genera are indicated: each defined cluster was supported by a significant bootstrap value (between 93 and 100). Only 2 viruses did not cluster in any of these 8 clades: a parvovirus identified in *Artibeus jamaicensis* fruit bat (Aj-Bt-PV-1, which we previously reported as the first member of a novel parvovirus genus, Canuti et al., 2011) and the Sl.L-PV-1. Both viruses are located between the *Dependoparvovirus* and the *Tetraparvovirus* genera and do not significantly cluster with any other known parvovirus, possibly representing 2 independent still undefined genera.

To better clarify the relationship between Sl.L-PV-1 and the most closely related parvoviruses, the amino acid identities of both NS1 and VP1 proteins within and between the clades were calculated (Table 1). Considering NS1 protein, the virus with the highest identity to Sl.L-PV-1 was the Bovine adeno-associated virus (BAAV, 29.1%), followed by other members of the *Dependoparvovirus* genus, which, on average, was the clade closest to Sl.L-PV-1. The *Tetraparvovirus* clade and Aj-Bt-PV-1 are approximately equidistant from Sl.L-PV-1. The same trend could be noted for Aj-Bt-PV-1.

**Table 1:** **Identities between and within the clades more closely related to Sl.L-PV-1**.

	**Dependoparvovirus**	**Tetraparvovirus**	**Aj-Bt_PV-1**	**Sl.L-PV-1**
Dependoparvovirus	*81.87 (61–45)*	17.21 (18.9–15.1)	21.01 (22.4–19.4)	23.4 (24.1–22.4)
	***43.27 (64.3–28)***			
Tetraparvovirus		*50.14 (72.9–27.3)*	18.8 (19.6–18.2)	20.4 (20.9–19.6)
	**21.18 (23.4–18.6)**	***46.45 (75.7–29.6)***		
Aj-Bt_PV-1			*id*	21.3
	**24.81 (26–22.4)**	**22.6 (23.1–22)**	***id***	
Sl.L-PV-1				*id*
	**26.6 (29.1–24.5)**	**22.8 (25–19.9)**	**23.6**	***id***

*Values represent average percentage identity (upper limit—lower limit) calculated using representative members of each species in the clades. Values in bold (bottom left) refers to NS1 amino acid sequence, while the other numbers (top right) refer to VP1 amino acid sequence. On the diagonal in italic are the identities within each clade; id, identical. The extended analysis is available in the Supplementary Tables 1, 2*.

The classification rules for parvoviruses state that a genus is defined as a monophyletic group in which the NS1 amino acidic sequences of all included viruses are generally more than 30% identical to each other and less than 30% identical to those of other genera (Cotmore et al., 2013). According to our analysis, both Aj-Bt-PV-1 and Sl.L-PV-1 could be considered the first members of 2 novel genera: their NS1 amino acid sequences are less than 30% identical to the ones of the viruses in the closest genera (Table 1), less than 25 and 27% respectively, and they are not part of a bootstrap supported monophyletic group (Figure 4).

### Screening and virus quantification

Twenty serum and 61 organ samples altogether belonging to 25 individuals (18 *N. coucang* and 7 *N. pygmaeus*) were screened for the presence of Sl.L-PV-1, but only samples collected from the animal in which the virus was originally identified were positive. All organs collected during necropsy were positive for the virus, with high viral loads as shown in Table 2. Liver, spleen, and kidneys were the organs where the virus reached the highest loads.

**Table 2:** **Sl.L-PV-1 loads determined in different organs (collected at necropsy in 2008) and from serum/blood samples collected during various years**.

**Material**	**Collection date (mm/dd/yyyy)**	**Viral load (copies/g or copies/ml)**
Lung	06/03/2008	6.09E + 07
Small intestine	06/03/2008	4.82E + 07
Large intestine	06/03/2008	1.24E + 07
Liver	06/03/2008	1.87E + 08
Spleen	06/03/2008	1.79E + 08
Kidney	06/03/2008	1.43E + 09
Serum	03/21/2000	1.90E + 07
Serum	12/08/2005	4.20E + 07
Whole blood	11/08/2007	5.39E + 08
Serum^*^	06/03/2008	1.08E + 06

* After VIDISCA pre-treatment (centrifugation and DNase treatment prior to DNA isolation)

Surprisingly, all blood/serum samples collected at different time points were PCR positive. Those samples were collected from Buddha on a time frame of 8 years (from the year 2000 until 2008) and the virus was constantly found at moderately high concentrations, with the highest titre found in whole blood (5.39E + 08 DNA copies/ml) 7 months prior to the death of the animal (Table 2).

### Virus evolution

A 1231 nt fragment of the VP1 ORF end was sequenced from all positive samples in order to detect variation between different body sites or among the 8 years of infection: 16 polymorphic sites were noted (Table 3). The polymorphisms predominantly included transitions (81.25%, 13/16) and only two were non-synonymous. If we consider only the sequences obtained from serum there is an accumulation of 5 substitutions (all transitions located between codons 405 and 536) during the years, one of which leads to an amino acid change. However, no fixation can be accounted if we consider altogether the sequences from 2008 (those retrieved both from serum and from all the organs).

**Table 3:** **Nucleotide polymorphisms in Sl.L-PV-1 identified over time and in different organs**.

**nt**	**3399**	**3443**	**3506**	**3530**	**3722**	**3746**	**3794**	**3923**
**AA**	**405 (V)**	**419 (L)**	**440 (Y)**	**448 (E)**	**512 (P)**	**520 (Q)**	**536 (E)**	**579 (T)**
Serum 2000	A (I)	T	T	G	T	A	A	Y
Serum 2005	R (V/I)	Y	Y	R	Y	R	R	Y
Whole blood 2007	R (V/I)	Y	Y	R	Y	R	R	Y
Serum 2008	G	C	C	G	C	R	G	C
Small intestine	R (V/I)	Y	Y	G	Y	A	R	C
Large intestine	G	Y	Y	G	Y	R	R	C
Lung	G	C	C	G	C	A	G	C
Spleen	G	C	C	G	Y	A	G	C
Liver	G	C	C	G	Y	A	G	C
Kidneys	R (IV/I)	Y	Y	R	Y	R	R	Y
**nt**	**3992**	**4040**	**4121**	**4331**	**4412**	**4475**	**4517**	**4558**
**AA**	**603 (S)**	**618 (R)**	**645 (F)**	**715 (G)**	**742 (S)**	**763 (T)**	**777 (V)**	**791 (S)**
Serum 2000	Y	R	T	T	R	M	Y	G
Serum 2005	Y	R	T	K	A	C	Y	K (S/I)
Whole blood 2007	Y	R	T	K	A	C	C	K (S/I)
Serum 2008	C	R	Y	K	A	C	C	K (S/I)
Small intestine	C	R	Y	K	R	M	Y	K (S/I)
Large intestine	Y	R	Y	K	A	C	C	K (I/S)
Lung	C	A	Y	K	A	C	C	K (I/S)
Spleen	Y	R	Y	K	A	C	C	K (I/S)
Liver	C	A	Y	K	A	C	C	K (I/S)
kidneys	Y	A	Y	K	A	C	C	K (I/S)

*Nucleotide positions refer to the whole genome as reported in GenBank (AN: KP120516); amino acids positions refer to the VP1 ORF. Where the mutation caused an amino acid change the new amino acid is indicated. Polymorphisms are indicated: Y (C or T), R (A or G), K (T or G), M (C or A)*.

The appearance of new variable sites over time can also be noted, like at codon position 645 where a polymorphism appeared only in 2008. Interesting to notice is that kidneys and, most of all, the intestine are the locations where most polymorphic sites characterizing earlier time points were conserved (positions 742, 763, and 777).

### Latent virus detection

Since the virus was detected in samples collected from Buddha during 8 years the presence of integrated or episomal viral genomes could be postulated. Besides, the disruption of an oncolytic gene caused by viral integration could have been at the origin of the HS and therefore the presence of integrated viral genomes in all tissues (including whole blood) was investigated by means of three different methods - since the integration location could not be predicted (Figure 1). No evidence for integrated viruses could be found. In addition, there was also no evidence of a circular covalently closed genomic form which could have persisted in tissues as an episomal form.

## Discussion

Cancer is one of the leading cause of human death worldwide and it represents a considerable health concern for domestic and wild animals as well as animals kept in captivity (WHO|Cancer^1^; Misdorp, 1996; Kelsey et al., 1998; McAloose and Newton, 2009; Remick et al., 2009). Although the full process of tumorigenesis is not completely understood, it is known that accumulation of mutations in proto-oncogenes (such as those involved in the regulation or suppression of cell replication or tumors) or the effect of viral infections are involved in the progression (Bergers and Benjamin, 2003; McAloose and Newton, 2009). The number of recognized oncogenic viruses is increasing and nowadays several examples—both from the human and animal fields—are well characterized. The best known examples are the papillomaviruses causing cervical cancers in humans and different types of genital and cutaneous cancers in aquatic mammals, the hepadnaviruses causing hepatocellular carcinomas in humans and woodchucks, the Epstein Barr virus which is responsible for Burkitt's lymphoma or retroviruses which are at the origin of different types of malignancies in humans and other mammals (McAloose and Newton, 2009; Braoudaki and Tzortzatou-Stathopoulou, 2011; Butt and Miggin, 2012; Pannone et al., 2014). It is therefore very important to identify novel oncoviruses and extensively study them in order to understand their tumorigenic mechanisms, especially in light of future developments such as prevention strategies.

In this study we investigated a case of diffuse histiocytic sarcoma—a rare but very aggressive type of hematopoietic tumor which can develop in different animal species (Fulmer and Mauldin, 2007; Friedrichs and Young, 2008; Soshin et al., 2008; Molenaar et al., 2009; Remick et al., 2009; Takahashi and Nakamura, 2013). To investigate the possible viral involvement in the etiopathogenesis of the HS a broad spectrum virus discovery technique was employed, which is able to detect virtually any DNA or RNA viruses present in a clinical sample (De Vries et al., 2011, 2012; Oude Munnink et al., 2014). A novel parvovirus was identified and molecularly fully characterized. The virus, which we named Slow Loris parvovirus 1 (Sl.L-PV-1), is around 5 kb in size and possesses all the molecular features typical of parvoviruses, including sequences coding for conserved enzymatic motifs and the 2 main ORFs, flanked by non-coding terminal regions, which appear identical but inverted like described for other parvoviruses (Lusby et al., 1980; Berns, 1990; Deiss et al., 1990). The virus is phylogenetically located between the *Dependoparvovirus* and the *Tetraparvovirus* genera and, according to the classification rules defined by the ICTV (Cotmore et al., 2013), is possibly the first member of a new genus.

Thanks to the availability of a series of serum and organ samples collected over multiple years from slow loris with various diseases, we were able to screen different samples from a total of 25 individuals belonging to the *N. coucang* species and to the closest related species *N. pygmaeus*. No other animal was positive for the virus but, as expected, all organs collected during the necropsy were positive, as also reported for other parvoviruses (Meunier et al., 1985; Canuti et al., 2011), and liver, spleen and kidneys were the organs where the virus reached the highest loads. The high viral concentrations found in these organs might reflect their elevated content of blood and blood cells. Besides, during the histological investigation the presence of basophilic inclusions in the renal tissue could be observed and these might represent parvoviral accumulations, similar to other reports for parvoviruses both *in vivo* (Hayes et al., 1979; Bestetti and Zwahlen, 1985; Porter et al., 1988; Decaro and Buonavoglia, 2012) and *in vitro* (Inaba et al., 1973; Oleksiewicz et al., 1996). Surprisingly, all blood or serum samples collected during various years were positive at a rather constant load: we could detect viremia 8 years prior to the death of the animal. Given the involvement in the disease of white blood cells, a presumed condition of immunosuppression (caused either by the virus, the tumor, or by a combination of these 2 factors) can be postulated and might be a possible explanation for the fact that the virus was not cleared in 8 years. The supposed presence of immunodeficiency in the loris is supported by the detection of opportunistic infections, as the pathological tests reported the presence of Toxoplasma gondii infection.

Since HS is a fast and very aggressive type of cancer we could have hypothesized that the virus was present in the Loris before tumor development. Histiocytes can refer to cells of either the macrophage or dendritic cell lineage as both arise from a common precursor cell. Neoplasms of histiocytes can arise from macrophages or one of two types of dendritic cells, Langerhans cells or interstitial dendritic cells. Langerhans cells are found within the epithelium and interstitial dendritic cells occupy a perivascular position in most tissues. Most forms of HS in animals arise as a malignant proliferation of interstitial dendritic cells, although there is one form, the hemophagocytic variant, that arises from macrophages (Fulmer and Mauldin, 2007; Takahashi and Nakamura, 2013; Moore, 2014). There is no established classification scheme for HS in prosimians. We interpreted this tumor to have most likely arisen from interstitial dendritic cells. Some parvoviruses have proven tropism for cells of the hematopoietic system and the bone marrow providing the perfect condition for parvoviral replication, since a wide spectrum of cells at different dividing stages are present and parvoviruses need actively dividing cells (S phase) to replicate (Berns, 1990; Segovia et al., 1991). In fact, hematopoietic disorders, like leucopenia or alterations in the bone marrow, can be observed in infected individuals (Larsen et al., 1976; Boosinger et al., 1982).

Although there is no formal proof of the existence of oncoparvoviruses, a causative link between the novel parvovirus and the HS could not be ruled out and therefore we investigated this hypothesis by exploring the eventual presence of latent forms of the virus. It is known that parvoviruses can establish latency by integrating in their hosts' genome (Schnepp et al., 2005; Kapoor et al., 2010), and an integration event might cause the disruption of important onco-suppressors and be one of the initial causes for tumor development. Episomal DNA forms of various parvoviruses have recently been detected (Kapoor et al., 2011; Zhao et al., 2012) and parvoviral DNA was found to persist in different tissues (Schneider et al., 2008; Norja et al., 2012), sometimes even in correlation with tumors (Li et al., 2012; Schildgen et al., 2013), although without a causative link. Since it was impossible to predict in which way the virus would have persisted, this hypothesis was tested with 4 different methods aiming at identifying either potentially integrated viral DNA or circular covalently closed genome persisting in tissues as episomal forms. None of these approaches gave evidence for latent forms of the virus in any of the tested organs (including whole blood).

A mutation hotspot located on the end side of the VP1 ORF was identified and an increase in variation during the years was observed. The evolutionary rates estimated in literature for parvoviruses are around 10E-4 substitutions per site per year in the studied genomic region (Shackelton and Holmes, 2006; Zehender et al., 2010; Streck et al., 2011) and the amount of substitutions we observed reflect those rates. However, these previously published rates have been estimated at an animal population level and not inter-host. In addition, fixation rates are connected to the transmission route of the virus: the sequences of the viral subpopulation which are transmitted to other individuals depend on the viral variation at the body site where the virus is shed. Since no other infected individual has been identified no postulations could be made on the transmission route of the virus and also no evolutionary rate estimations were possible. Nevertheless, the observed increase in sequence variation over time is inconsistent with a latency hypothesis: an integrated virus would evolve at the same evolutionary rate as its host and therefore, in case of integration, no variation would be observed.

It is reasonable to believe that the Sl.L-PV-1 was not the cause of the malignancy but merely found the ideal replication condition in cancer cells since, as discussed above, it is well known that parvoviruses need actively replicating cells (S-phase) in order to proliferate (Berns, 1990). In fact it is known that certain viruses, called oncolytic viruses, have tropism for specific cancer cells and they can even lead to “spontaneous regression” of malignancies (Butt and Miggin, 2012; Sze et al., 2013). This has been proven for the adeno-associated *dependoparvovirus*, although in an indirect way by enhancing adenoviral replication, and for a rodent parvovirus (Nüesch et al., 2012; Laborda et al., 2013). Future research will have to clarify whether this viral infection precedes tumor development and has a role in the oncogenic process, whether the virus simply benefits from the replication activity of the cancer cells or if the persistent viremia has no correlation with the HS. An answer to this question will be obtained by identifying the cell types where viral replication occurs after the detection of other infected loris and the constant monitoring of their physical condition and disease progression, and after the discovery of related viruses in other animals with similar malignancies. Although more studies are required to provide a conclusive answer, the infection with Sl.L-PV-1 might have delayed tumor progression. If this mechanism will be proven feasible new exciting possibilities might open for oncolytic parvovirotherapy (Nüesch et al., 2012; Russell et al., 2012).

In conclusion we discovered and molecularly characterized a novel parvovirus, the first member of a not yet defined genus and the first described in prosimians. The virus was identified in a slow loris with HS but we found no evidence for a causative involvement in the neoplastic disease and postulated that the virus had a replication advantage derived from the constantly replicating cancer cells.

### Conflict of interest statement

The authors declare that the research was conducted in the absence of any commercial or financial relationships that could be construed as a potential conflict of interest.

## Footnotes

^1^http://www.who.int/mediacentre/factsheets/fs297/en/ [Accessed May 19, 2014].
