# Supplementary material for: Persistent viremia by a novel parvovirus in a slow loris (Nycticebus coucang) with diffuse histiocytic sarcoma
Source: Front Microbiol. 2014 Dec 1;5:655. doi: 10.3389/fmicb.2014.00655 (PMC4249460; doi:10.3389/fmicb.2014.00655)
Supplement: Supplementary file 1 [file Table1.DOCX]

Persistent viremia by a novel parvovirus in a slow loris (*Nycticebus coucang*) with diffuse histiocytic sarcoma

Marta Canuti^1,*,#^, Cathy V. Williams^2^, Sashi Rekha Gadi^3^, Maarten F. Jebbink^1^, Bas B. Oude Munnink^1^, Seyed Mohammad Jazaeri Farsani^1,4^, John Cullen^3^, Lia van der Hoek^1,*^

1. Laboratory of Experimental Virology, Department of Medical Microbiology, Center for Infection and Immunity Amsterdam (CINIMA), Academic Medical Center of the University of Amsterdam, Amsterdam, the Netherlands.

2. Duke Lemur Center, Durham, NC, USA

3. College of Veterinary Medicine, North Carolina State University, Raleigh, NC, USA

4. Tehran University of Medical Sciences, Tehran, Iran

**Supplementary table 1: identities between NS1 amino acid sequences of viruses belonging to the *Dependoparvovirus* genus, *Tetraparvovirus* genus, Aj-Bt_PV-1 and Sl.L-PV-1 used to calculate averages values showed in Table 1.**

|  | AAV2 | CslAAV | BAAV | GPV | AAAV | BtAAV | SAAV | Ba-PARV4 | PARV4 G1 | B-PA RV4-1 | P-PA RV4 | CnP-PARV4 | O-PA RV4 | Aj-Bt PV1 |
| --- | --- | --- | --- | --- | --- | --- | --- | --- | --- | --- | --- | --- | --- | --- |
| CslAAV | 0.547 |  |  |  |  |  |  |  |  |  |  |  |  |  |
| BAAV | 0.573 | 0.643 |  |  |  |  |  |  |  |  |  |  |  |  |
| GPV | 0.433 | 0.452 | 0.456 |  |  |  |  |  |  |  |  |  |  |  |
| AAAV | 0.523 | 0.439 | 0.452 | 0.409 |  |  |  |  |  |  |  |  |  |  |
| BtAAV | 0.499 | 0.496 | 0.48 | 0.412 | 0.428 |  |  |  |  |  |  |  |  |  |
| SAAV | 0.321 | 0.309 | 0.321 | 0.28 | 0.301 | 0.312 |  |  |  |  |  |  |  |  |
| Ba-PARV4 | 0.224 | 0.214 | 0.215 | 0.216 | 0.227 | 0.234 | 0.201 |  |  |  |  |  |  |  |
| PARV4G1 | 0.21 | 0.214 | 0.215 | 0.216 | 0.217 | 0.213 | 0.199 | 0.394 |  |  |  |  |  |  |
| B-PARV4-1 | 0.207 | 0.21 | 0.214 | 0.213 | 0.204 | 0.213 | 0.2 | 0.409 | 0.564 |  |  |  |  |  |
| P-PARV4 | 0.22 | 0.224 | 0.225 | 0.221 | 0.214 | 0.221 | 0.197 | 0.423 | 0.548 | 0.671 |  |  |  |  |
| CnP-PARV4 | 0.204 | 0.206 | 0.196 | 0.207 | 0.208 | 0.208 | 0.186 | 0.296 | 0.313 | 0.305 | 0.322 |  |  |  |
| O-PARV4 | 0.213 | 0.216 | 0.211 | 0.218 | 0.21 | 0.219 | 0.195 | 0.415 | 0.563 | 0.757 | 0.682 | 0.306 |  |  |
| Aj-BtPV1 | 0.243 | 0.258 | 0.251 | 0.25 | 0.26 | 0.251 | 0.224 | 0.22 | 0.227 | 0.227 | 0.222 | 0.231 | 0.229 |  |
| Sl.L-PV-1 | 0.276 | 0.288 | 0.291 | 0.248 | 0.257 | 0.261 | 0.245 | 0.213 | 0.25 | 0.234 | 0.236 | 0.199 | 0.236 | 0.236 |

GenBank accession Number list:

*Dependoparvovirus*:

*Adeno-associated dependoparvovirus A:* AAV2, AF043303

*Adeno-associated dependoparvovirus B*: BAAV, AY388617

*Anseriform dependoparvovirus 1*: GPV, U25749

*Avian dependoparvovirus 1*: AAAV, AY186198

*Chiropteran dependoparvovirus 1*: BtAAV, GU226971

*Pinniped dependoparvovirus 1*: CslAAV, JN420372

*Squamate dependoparvovirus 1*: SAAV, AY349010

*Tetraparvovirus*:

*Chiropteran tetraparvovirus 1*: Ba-PARV4, JQ037753

*Primate tetraparvovirus 1*: PARV4G1, AY622943

*Ungulate tetraparvovirus 1*: B-PARV4-1, EU200669

*Ungulate tetraparvovirus 2*: P-PARV4, EU200677

*Ungulate tetraparvovirus 3*: CnP-PARV4, GU938300

*Ungulate tetraparvovirus 4*: O-PARV4, JF504699

Unassigned parvoviruses:

*Artibeus jamaicensis* bat parvovirus 1: Aj-BtPV1, JQ037754

Slow loris parvovirus 1: Sl.L-PV-1, KP120516

**Supplementary table 2: identities between VP1 amino acid sequences of viruses belonging to the *Dependoparvovirus* genus, *Tetraparvovirus* genus, Aj-Bt_PV-1 and Sl.L-PV-1 used to calculate averages values showed in Table 1.**

|  | AAV2 | CslAAV | BAAV | GPV | AAAV | BtAAV | SAAV | Ba- PARV4 | PARV4 G1 | B-PA RV4-1 | P-PA RV4 | CnP-PA RV4 | O-PA RV4 | Aj-BtPV1 |
| --- | --- | --- | --- | --- | --- | --- | --- | --- | --- | --- | --- | --- | --- | --- |
| CslAAV | 0.512 |  |  |  |  |  |  |  |  |  |  |  |  |  |
| BAAV | 0.568 | 0.61 |  |  |  |  |  |  |  |  |  |  |  |  |
| GPV | 0.505 | 0.488 | 0.49 |  |  |  |  |  |  |  |  |  |  |  |
| AAAV | 0.553 | 0.493 | 0.524 | 0.557 |  |  |  |  |  |  |  |  |  |  |
| BtAAV | 0.606 | 0.517 | 0.56 | 0.516 | 0.527 |  |  |  |  |  |  |  |  |  |
| SAAV | 0.485 | 0.45 | 0.463 | 0.501 | 0.462 | 0.506 |  |  |  |  |  |  |  |  |
| Ba-PARV4 | 0.162 | 0.158 | 0.165 | 0.171 | 0.159 | 0.174 | 0.176 |  |  |  |  |  |  |  |
| PARV4G1 | 0.17 | 0.176 | 0.176 | 0.182 | 0.172 | 0.189 | 0.176 | 0.504 |  |  |  |  |  |  |
| B-PARV4-1 | 0.174 | 0.171 | 0.176 | 0.183 | 0.169 | 0.189 | 0.169 | 0.491 | 0.658 |  |  |  |  |  |
| P-PARV4 | 0.176 | 0.162 | 0.168 | 0.183 | 0.17 | 0.184 | 0.179 | 0.507 | 0.651 | 0.661 |  |  |  |  |
| CnP-PARV4 | 0.16 | 0.151 | 0.159 | 0.165 | 0.151 | 0.173 | 0.165 | 0.273 | 0.307 | 0.298 | 0.314 |  |  |  |
| O-PARV4 | 0.175 | 0.172 | 0.182 | 0.183 | 0.17 | 0.185 | 0.178 | 0.5 | 0.676 | 0.729 | 0.662 | 0.29 |  |  |
| Aj-BtPV1 | 0.205 | 0.194 | 0.212 | 0.224 | 0.212 | 0.218 | 0.206 | 0.188 | 0.184 | 0.19 | 0.196 | 0.187 | 0.182 |  |
| Sl.L-PV-1 | 0.235 | 0.229 | 0.232 | 0.24 | 0.224 | 0.235 | 0.241 | 0.207 | 0.209 | 0.204 | 0.205 | 0.196 | 0.205 | 0.213 |

GenBank accession Number list:

*Dependoparvovirus*:

*Adeno-associated dependoparvovirus A:* AAV2, AF043303

*Adeno-associated dependoparvovirus B*: BAAV, AY388617

*Anseriform dependoparvovirus 1*: GPV, U25749

*Avian dependoparvovirus 1*: AAAV, AY186198

*Chiropteran dependoparvovirus 1*: BtAAV, GU226971

*Pinniped dependoparvovirus 1*: CslAAV, JN420372

*Squamate dependoparvovirus 1*: SAAV, AY349010

*Tetraparvovirus*:

*Chiropteran tetraparvovirus 1*: Ba-PARV4, JQ037753

*Primate tetraparvovirus 1*: PARV4G1, AY622943

*Ungulate tetraparvovirus 1*: B-PARV4-1, EU200669

*Ungulate tetraparvovirus 2*: P-PARV4, EU200677

*Ungulate tetraparvovirus 3*: CnP-PARV4, GU938300

*Ungulate tetraparvovirus 4*: O-PARV4, JF504699

Unassigned parvoviruses:

*Artibeus jamaicensis* bat parvovirus 1: Aj-BtPV1, JQ037754

Slow loris parvovirus 1: Sl.L-PV-1, KP120516
